# Correction: Mapping brain-behavior space relationships along the psychosis spectrum

**DOI:** 10.7554/eLife.79129

**Published:** 2022-04-05

**Authors:** Jie Lisa Ji, Markus Helmer, Clara Fonteneau, Joshua B Burt, Zailyn Tamayo, Jure Demšar, Brendan D Adkinson, Aleksandar Savić, Katrin H Preller, Flora Moujaes, Franz X Vollenweider, William J Martin, Grega Repovš, Youngsun T Cho, Christopher Pittenger, John D Murray, Alan Anticevic

**Keywords:** Human

Ji JL, Helmer M, Fonteneau C, Burt JB, Tamayo Z, Demšar J, Adkinson BD, Savić A, Preller KH, Moujaes F, Vollenweider FX, Martin WJ, Repovš G, Murray JD, Anticevic A. 2021. Mapping brain-behavior space relationships along the psychosis spectrum. *eLife* **10**:e66968. doi: 10.7554/eLife.66968.

A supplemental analysis in the final paper involved an independent replication dataset. This replication dataset describes a sub-sample of patients diagnosed with Obsessive Compulsive Disorder (OCD) and schizophrenia which was collected as part of a larger project in collaboration with Dr. Chris Pittenger. In addition, Dr. Youngsun T. Cho played a key role in coordinating the data collection and management of this project. We are therefore formally correcting the eLife paper to include both Dr. Pittenger and Dr. Cho as co-authors on this paper. Both have reviewed the manuscript and support the conclusions, and agree to be responsible for all parts of the paper in its published form.

The details of Dr. Pittenger and Dr. Cho’s contributions are explained below:

Dr. Pittenger and Dr. Cho provided key support in the data acquisition and coordination of the project from which the independent replication dataset was obtained. This replication dataset involved a subsample of data with OCD and schizophrenia that were collected as part of a larger study in collaboration with Dr. Pittenger. Dr. Cho was also integral for the project management of this study. Both Dr. Pittenger and Dr. Cho have reviewed the manuscript and its conclusions.

**New authors list:**

Jie Lisa Ji, Markus Helmer, Clara Fonteneau, Joshua B Burt, Zailyn Tamayo, Jure Demšar, Brendan D Adkinson, Aleksandar Savić, Katrin H Preller, Flora Moujaes, Franz X Vollenweider, William J Martin, Grega Repovš, Youngsun T. Cho, Christopher Pittenger, John D Murray, Alan Anticevic

**Original authors list:**

Jie Lisa Ji, Markus Helmer, Clara Fonteneau, Joshua B Burt, Zailyn Tamayo, Jure Demšar, Brendan D Adkinson, Aleksandar Savić, Katrin H Preller, Flora Moujaes, Franz X Vollenweider, William J Martin, Grega Repovš, John D Murray, Alan Anticevic

Details for the omitted authors:

Youngsun T. Cho

Department of Psychiatry, Yale University School of Medicine, New Haven, United States; Child Study Center, Yale University School of Medicine, New Haven, United States.

**Contribution:** Conceptualization, Resources, Data curation, Supervision, Funding acquisition, Investigation, Methodology

**Competing Interests:** YC declares no competing interests.

Christopher Pittenger

Department of Psychiatry, Yale University School of Medicine, New Haven, United States; Child Study Center, Yale University School of Medicine, New Haven, United States.

**Contribution:** Conceptualization, Resources, Data curation, Supervision, Funding acquisition, Investigation, Methodology

 CP has consulted in the past 3 years to Biohaven Pharmaceuticals, Lundbeck Parmaceuticals, Ceruvia Therapeutics, Transcend Therapetics, Freedom Biosciences, Teva Pharmaceuticals and Brainsway Therapeutics and receives research funding from Biohaven Pharmaceuticals, the Usona Institute, and Transcend Pharmaceuticals. [1] None of these sources of support are related to the present work. CP is an inventor on two pending patent applications, 16/304,925 and 63/074,275, neither of which is relevant to the current work.

The article has been corrected accordingly.

